# Recollections of John Fox: One of the founders of medical AI

**DOI:** 10.1002/lrh2.10308

**Published:** 2022-03-15

**Authors:** Jeremy C. Wyatt

**Affiliations:** ^1^ Faculty of Medicine University of Southampton Southampton UK

John Fox, who died in 2021, was a productive, far sighted cognitive and AI scientist best known for his work on the Oxford System of Medicine, the Proforma guideline modelling language and the OpenClinical computable knowledge publishing model.

The Oxford System of Medicine (OSM) was a pioneering primary care decision support tool developed in the late 1980s in collaboration with Oxford University Press, supported by three British GPs (Mike O'Neill, Andrzej Glowinski and Peter Pritchard).^1^ This 5‐year project explored how to develop a large scale, maintainable knowledge base with many thousands of declarative facts and a handful of generic rules that operated over them. OSM was initially developed using PROPS‐2, a Prolog‐based inference engine developed in the late 1980's by Saki Hajnal and other members of John's team at the Imperial Cancer Research Fund (ICRF—now merged into Cancer Research UK) in London. PROPS‐2 was a well‐supported expert system toolkit with a user manual and user group that was widely disseminated and used for prototyping many knowledge‐based systems in the early years of medical AI, such as ACORN.^2^

The OSM provided a starting point for the development of John's ideas on symbolic decision procedures for autonomous agents (including human agency) that were taken forward by members of his Advanced Computation Laboratory at ICRF. Colin Gordon was the academic lead for the sequence of EU projects that took the OSM decision model and applied it to the clinical decisions involved in managing cancer treatments,^3^ which evolved into a framework for representing clinical guidelines. John worked with Paul Krause,^4^ Simon Parsons^5^ and others at the ACL to develop the foundational models for the logical assessment of arguments for and against different courses of action. This was early work in a strand that has now grown into a significant international body of academic research on argumentation. His ideas diverged from the mainstream data‐driven approaches that have gained so much prominence in the last three decades, but anticipated concerns about interpretability and safety which are increasingly at the forefront of thinking about AI.

One outcome of this research was the development of PROFORMA, a language for modelling computable guidelines based on a rigorous logic‐based argumentation approach.^6^, ^7^ It is a symbolic decision and planning language which provides an expressive formalism for modelling professional expertise and deploying ‘human friendly’ decision services and other cognitive agents, forming part of Fox's CREDO framework.^8^ Graphical programming tools allow the user to capture most of the logical structures found in practice guidelines in executable form, and PROFORMA has been tested in many clinical areas with 16 published studies (some of which are randomised trials) showing clinical benefit.^9^

The PROFORMA toolkit and language are complemented by a novel open publishing model that starts by developing a PROFORMA ‘publet’ (computable guideline fragment), documenting it and placing the results on the OpenClinical website^9^ for open peer review and revision. OpenClinical is a not‐for‐profit community interest company founded in 2003, aimed at improving patient care by promoting a new publication model for innovative AI and knowledge engineering techniques. It provides incubator and trialling services for machine executable models of clinical decision‐making and workflow management using the OpenClinical platform, and is closely aligned with our current interest in Mobilising Computable Biomedical Knowledge, MCBK.^10^ OpenClinical has recently been spun‐in to Oxford University Innovation's portfolio of not‐for‐profit companies—see www.openclinical.net. There are currently over 50 publets available for anyone to use on the site, including a recent COVID management guideline described in *Learning Health Systems* journal.^11^

John Fox also founded a learned journal in 1984, the Knowledge Engineering Review, and was editor for 12 years. This journal started with thin glossy paper and few subscribers but later gained the support of Cambridge University Press, has become widely read and cited and is now in its 36th volume.^12^ John was also influential in the formation of the British Computer Society Expert System group and the European Society for AI in Medicine in the 1980s and trained many PhDs and post docs, some of whom (such as Enrico Coiera and Paul Taylor) are now leading the discipline.

John described himself as “Researcher in the scientific foundations, practical technologies and deployment of AI and cognitive systems for the real world.”^13^ After a PhD in psychology in Cambridge where he was supervised by Donald Broadbent FRS, he spent 2 years as a postdoc in the USA working at CMU with AI founders Allen Newell and Herb Simon and then at Cornell before returning to a post with the Medical Research Council in Sheffield.

I first met John in 1985 soon after he had set up the Advanced Computation Lab and was advertising for two doctors to join him for the OSM project. In 1981, he had persuaded Sir Walter Bodmer FRS, then Director of ICRF, to remove his programming group from the computational support unit for ICRF scientists to become a new research unit, the Advanced Computation Lab (ACL). Spurred on by a series of quinquennial reviews carried out by distinguished international scientists, John built up the ACL over 25 years to become a large, productive research unit, collaborating with a range of EU Framework projects (Richard Thompson managed most of the complexity of these), before moving to the Department of Engineering Science in Oxford in 2007 to become Professor of Cognitive Systems Engineering with Sir Michael Brady.

John also co‐founded several successful medical AI start‐ups including Expertech, InferMed and Deontics, at least one of which originated in an EU Framework project. InferMed received the Queen's Award for Innovation and Enterprise in 2012 and was acquired by the medical and scientific publisher Elsevier in 2015. He had an international profile with nearly 5000 LinkedIn followers and gave numerous invited keynote talks. Among his other achievements was an early book about the safety of AI published in 2000 with Subrata Das,^14^ and many scientific articles on the nature of reasoning and different methods for supporting this. His H index was nearly 50.

John made a very significant contribution to our field and his influence will continue to be felt for many years. Many of us feel privileged to have known and worked with him. While we did not always agree with his approaches and thinking, he was always thought provoking, generous, knowledgeable and committed. His practical tools such as PROPS‐2 and PROFORMA and theoretical work such as CREDO have provided strong foundations for the medical AI field.^8^ In recognition of his contributions, the European Society for AI in Medicine, AIME, has agreed to include a John Fox Memorial Lecture in its biennial conferences.
